# Developing Preoperative Nomograms to Predict Any-Stage and Stage III-IV Endometriosis in Infertile Women

**DOI:** 10.3389/fmed.2020.570483

**Published:** 2020-10-22

**Authors:** Zaixin Guo, Penghui Feng, Xiaohan Chen, Ruiyi Tang, Qi Yu

**Affiliations:** Department of Obstetrics and Gynecology, Peking Union Medical College Hospital, Peking Union Medical College and Chinese Academy of Medical Sciences, Beijing, China

**Keywords:** endometriosis, infertility, logistic regression, nomogram, predictive model

## Abstract

**Study objective:** To generate and validate nomograms to predict any-stage and stage III-IV endometriosis before surgery in infertile women.

**Design:** A single center retrospective cohort study.

**Setting:** University affiliated hospital.

**Patients:** Infertile patients (*n* = 1,016) who underwent reproductive surgery between July 1, 2016 and June 30, 2019.

**Interventions:** None.

**Main outcome measurements:** We randomly selected 2/3 of the included patients (667 patients, training sample) to analyze and generate predictive models and validated the models on the remaining patients (339 patients, validation sample). A multivariate logistic regression model was used with the training sample to select variables using a back stepwise procedure. Nomograms to predict any-stage and stage III–IV endometriosis were constructed separately. The discriminations and calibrations of both nomograms were tested on the overall population and a subgroup without endometrioma diagnosed on transvaginal sonography (TVS) of training and validation samples. The impact of different variables in these models was evaluated.

**Results:** There were 377 (55.7%) women in the training sample and 196 (57.8%) in the validation sample who were diagnosed with endometriosis. The nomogram predicting any-stage endometriosis had an area under the curve (AUC) of 0.760 for the training sample and 0.744 for the validation sample, with favorable calibrations in the overall population. However, the performance was significantly decreased in patients without endometrioma on TVS, with an AUC of 0.726 in the training sample and 0.694 in the validation sample. Similarly, the nomogram predicting stage III–IV endometriosis had an AUC of 0.833 and 0.793 for the training and validation samples, respectively, as well as a favorable calibration. However, the performance of the nomogram on patients without endometrioma on TVS was poor. Endometrioma on TVS strongly predicted both any stage and stage III–IV endometriosis on both samples.

**Conclusion:** We developed nomograms to predict any-stage and stage III–IV endometriosis but their performance were significantly decreased in patients without endometrioma on TVS. Endometrioma on TVS strongly predicted any and III–IV stage endometriosis in both sample groups. Therefore, we recommend that this study be used as encouragement to advance the utilization of advanced imaging for endometriosis for better clinical prognosis.

## Introduction

Endometriosis is a common gynecological disorder characterized by the presence of endometrial tissue outside of the uterine cavity (1). Women with endometriosis typically present with infertility and pelvic pain, but can be asymptomatic (2). Among infertile women, the prevalence of endometriosis is 25–50% (3). To make a definitive diagnosis of endometriosis, laparoscopic inspection of the pelvis is necessary (4). However, as *in vitro* fertilization (IVF) is an alternative choice for infertile women, it will be helpful if endometriosis is identified with preoperative clinical data (5).

Plenty of studies have associated preoperative clinical data with endometriosis (6). However, no consensus has been reached on the best predictive models for endometriosis due to the diverse study population, different diagnostic criteria, and various predictive factors (6, 7). The most common models using pain to predict endometriosis are not as accurate in diagnosing asymptomatic endometriosis, which is often found in infertile women (8). Several reports have predicted endometriosis in infertile women yet with some limitations. Some studies use history and symptoms as predictive factors, generating areas under the curve (AUC) of 0.71 and 0.752 (5, 9). Some studies are not practical, as they used patients with normal pelvis as controls (10, 11).

We aimed to build simplified nomograms to preoperatively predict any-stage and stage III-IV endometriosis in infertile women. Hysterosalpingography (HSG) findings specific to infertility were incorporated into the models. A subgroup of patients without endometrioma was used to illustrate the performance of two nomograms. In addition, we altered the variables in the full models for a better understanding of their effect for any-stage and stage III–IV endometriosis.

## Materials and Methods

This is a single center retrospective cohort study, which was approved by the Ethics Committee of Peking Union Medical College Hospital.

### Patients

**Figure 1:**
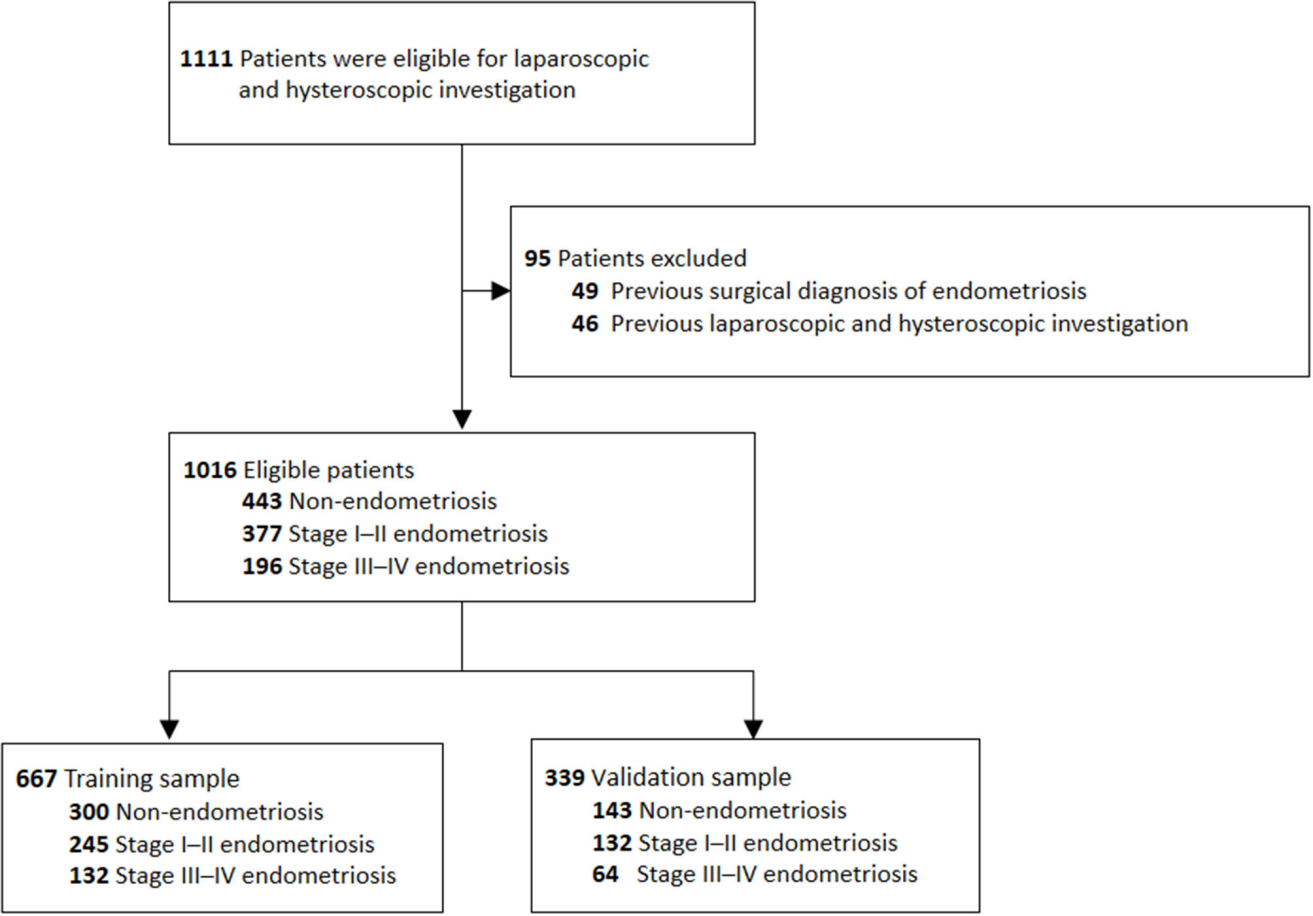
Flow chart for establishing of the training and validation sample. A total of 1,111 patients underwent laparoscopic and hytseroscopic investigation at our hospital, but 95 patients were excluded from the study due to a history of endometriosis or previous laparoscopic or hysteroscopic investigations. Of the remaining 1,016 patients, 667 patients were allocated to the training sample and 339 patients were allocated to the validation sample.

The inclusion criteria were infertile patients who underwent reproductive surgery by laparoscopy and hysteroscopy for infertility (defined as attempting pregnancy for ≥1 year without success) in the Gynecological Endocrine and Reproduction Center of Peking Union Medical College Hospital (Beijing, China) between July 1, 2016 and June 30, 2019. The exclusion criteria were patients with a previous surgical diagnosis of endometriosis, or previous laparoscopic or hysteroscopic investigations. Figure 1 shows the patient selection flowchart. Endometriosis was diagnosed using laparoscopy on visual evidence alone according to the European Society of Human Reproduction and Embryology guidelines (4). Endometriosis was scored using the revised American Fertility Society (r-AFS) classification system and classified into four stages: I (minimal), II (mild), III (moderate), or IV (severe) (12). Patients who were not diagnosed with endometriosis (with or without other diagnoses) at laparoscopy were used as the controls for this study.

### Data Collection and Variable Definitions

The data were gathered by trained doctors who reviewed the patients' medical records. The infertility investigations performed in our center include clear medical records and standard reproductive surgeries. The standard infertility investigation includes a female medical history, symptoms, bimanual pelvic examination, ultrasound findings, and blood analysis of reproductive hormones and thyroid function, as well as a male medical history and sperm analysis. Most patients who do not have a clear indication for surgery undergo HSG. Once all exams and procedures have been performed, patients are counseled regarding IVF or a hysteroscopic and laparoscopic investigation. Surgery is recommended to investigate for possible endometriosis in women with normal ovulation and tubal patency whose partners have a normal semen. In this study, all surgeries were performed and recorded by gynecological surgeons. Procedures were performed under general anesthesia, and endometriosis was scored and staged by a visual inspection of the abdomen and pelvis. All recognizable endometriotic lesions were radically excised to reconstruct the pelvic anatomy whenever achievable without affecting fertility.

Variables with both positive and negative results in all patients were included in the study. Duration of infertility was defined as the period between the time the couple had started trying to conceive and the time of surgery. Pain was defined as dysmenorrhea with need of analgesic medication most of the time, and/or intermenstrual pelvic pain, and/or dyspareunia. Palpable nodularities in the pouch of Douglas were found on bimanual pelvic examination (13). Endometrioma was defined as the presence of a cyst or multiple cysts containing diffuse low-level echoes in the ovaries on transvaginal sonography (TVS) (14). Tubal pathologies diagnosed by HSG were classified as no tubal occlusion, distal tubal occlusion, or proximal tubal occlusion (defined as contrast not shown beyond the isthmic portion of the tube) (15). For patients without HSG results, tubal testing findings at laparoscopy were used as alternatives.

### Statistical Analyses

A random sample of two-thirds of the included patients was obtained prior to the analysis to develop a clinical prediction model (training sample), leaving one-third of the patients for validation (validation sample).

We first compared the baseline characteristics in patients without endometriosis, those with endometriosis stage I–II, and those with endometriosis stage III–IV using a chi-square test or an analysis of variance (ANOVA), where appropriate. We adjusted for the following covariates based on confounding relationships: BMI (<18.4, 18.5–22.4, 22.5–24.9, and ≥25 kg/m^2^) (16), duration of infertility (<2, 2–3, and >3 years)(9), age at menarche (<12, 12–13, and ≥14 years)(17), menstrual cycle length (≤24, 25–29, 30–34, and ≥35 days) (17), and length of natural menses (≤4, 5–6, and ≥7) (17).

The endpoints of the study were any-stage and stage III–IV endometriosis. Multivariable logistic regression analysis was used to select the best combination of variables that was independently associated with the diagnoses. Variables were selected by a backward, stepwise procedure. The *P*-values in the multivariable analysis were based on Wald tests. A *P*-value of 0.05 was considered significant. The final model equations were organized as nomograms designed to calculate patient-specific probabilities of any-stage or stage III–IV endometriosis. The models were applied to the validation sample and a subgroup of patients without endometrioma diagnosed on TVS. The discrimination of the models was assessed by AUC. A 95% confidence interval (95% CI) was calculated for the AUC. The differences in the AUCs between our models were compared using DeLong's test (18). The calibration of the models was assessed by calibration curves (19). We evaluated the *P*-value of unreliability statistic, average, and maximal errors between predictions and observations obtained from a calibration curve (19).

To better understand the difference between the two models, we built a model that included all of the variables (patient history and symptom, palpable nodularity, endometrioma diagnosed on TVS, and tubal pathology), and investigated how the predictive performance changed when each variable was removed. Additionally, a model with endometrioma diagnosed only on TVS was also built. The predictive performance of each model was determined by AUCs, which were compared using DeLong's test. Comparisons of discrimination and reclassification performance between the models were evaluated by calculating the integrated discrimination improvement (IDI) (20).

All analyses were performed using the R package with rms, pROC, Hmisc, and CalibrationCurves libraries (http://lib.stat.cmu.edu).

## Results

Overall, 1,111 patients met the inclusion criteria, however, 49 patients were excluded due to a previous surgical diagnosis of endometriosis, and 45 patients were excluded due to previous laparoscopic and hysteroscopic investigations. Of the remaining 1,016 patients, 443 patients (43.6%) did not have endometriosis, and 573 patients (56.4%) had visual endometriosis.

The training sample included 667 randomly selected patients: 300 patients without endometriosis, 245 patients with stage I–II endometriosis, and 132 patients with stage III–IV endometriosis. The validation sample included 339 patients: 142 patients without endometriosis, 132 patients with stage I–II endometriosis, and 64 patients with stage III–IV endometriosis. Patient characteristics are summarized in Table 1. There were no significant differences in the patient characteristics between the groups.

**Table 1:** Characteristics of the validation and training samples.

	**Training sample (*N* = 677)**	**Validation sample (*N* = 339)**	***P*-value**
Diagnosis- *n* (%)			0.69
Non-endometriosis	300 (44.3)	143 (42.2)	
Stage I or II endometriosis	245 (36.2)	132 (38.9)	
Stage III or IV endometriosis	132 (19.5)	64 (18.9)	
Age (years)-mean (SD)	32.81 (4.02)	32.79 (4.12)	0.93
BMI-*n* (%)			0.59
<18.4 kg/m^2^	72 (10.6)	31 (9.1)	
18.5–22.4 kg/m^2^	369 (54.5)	185 (54.6)	
22.5–24.9 kg/m^2^	139 (20.5)	80 (23.6)	
≥25 kg/m^2^	97 (14.3)	43 (12.7)	
Duration of infertility-*n* (%)			0.68
<2 years	147 (21.7)	71 (20.9)	
2–3 years	315 (46.5)	151 (44.5)	
>3 years	215 (31.8)	117 (34.5)	
Gravida-*n* (%)			0.69
0	397 (58.6)	204 (60.2)	
≥1	151 (22.3)	75 (22.1)	
Parity-*n* (%)			0.26
0	632 (93.4)	309 (91.2)	
≥1	45 (6.6)	30 (8.8)	
Menarche-*n* (%)			0.55
<12 years	40 (5.9)	16 (4.7)	
12–13 years	392 (57.9)	207 (61.1)	
≥14 years	245 (36.2)	116 (34.2)	
Cycle length-*n* (%)			0.51
≤ 24 days	14 (2.1)	6 (1.8)	
25–29 days	328 (48.4)	152 (44.8)	
30-34 days	235 (34.7)	134 (39.5)	
≥35 days	100 (14.8)	47 (13.9)	
Length of menstruation-*n* (%)			0.98
≤ 4 days	73 (10.8)	38 (11.2)	
5–6 days	393 (58.1)	196 (57.8)	
≥7 days	211 (31.2)	105 (31.0)	
Pain^a^ -*n* (%)	137 (20.2)	59 (17.4)	0.32
Palpable nodularity^b^ -*n* (%)	69 (10.2)	43 (12.7)	0.28
Endometrioma diagnosed on TVS-*n* (%)	81 (12.0)	40 (11.8)	1.0
Tubal pathology diagnosed on HSG-*n* (%)	581 (85.8)	291 (85.8)	1.0
Tubal pathology-*n* (%)			0.41
Bilateral normal	407 (60.1)	206 (60.8)	
Unilateral proximal tubal occlusion	69 (10.2)	32 (9.4)	
Bilateral proximal tubal occlusion	22 (3.2)	9 (2.7)	
Unilateral distal tubal occlusion	97 (14.3)	39 (11.5)	
Unilateral distal tubal occlusion, contra-lateral proximal tubal occlusion	27 (4.0)	13 (3.8)	
Bilateral distal tubal occlusion	55 (8.1)	40 (11.8)	

*BMI, body mass index; SD, Standard Deviation; TVS, transvaginal sonography*.

a *Presence of dysmenorrhea with need of analgesic medication most of the time, and/or intermenstrual pelvic pain, and/or dyspareunia*.

b *Presence of a palpable nodularity in pouch of Douglas on bimanual examination*.

The characteristics of the patients without endometriosis, with stage I–II endometriosis, and with stage III–IV endometriosis from the training sample are summarized in Table 2. BMI (*P* = 0.005), gravida (*P* = 0.031), parity (*P* = 0.029), menstrual cycle length (*P* = 0.033), length of menstruation (P = 0.008), pain (*P* < 0.001), palpable nodularity (*P* < 0.001), endometrioma diagnosed on TVS (*P* < 0.001), and tubal pathology (*P* < 0.001) were all significantly different between the three subgroups.

**Table 2:** Characteristics and diagnostic test results associated with no endometriosis, stage I–II endometriosis, and stage III–IV in the training sample.

	**No endometriosis (*N* = 300)**	**Stage I–II endometriosis (*N* = 245)**	**Stage III–IV endometriosis (*N* = 132)**	***P*-value**
Age (years)-mean (SD)	32.72 (4.44)	32.94 (3.76)	32.77 (3.45)	0.809
BMI-n (%)				0.005
<18.4 kg/m^2^	22 (7.3)	36 (14.7)	14 (10.6)	
18.5–22.4 kg/m^2^	157 (52.3)	130 (53.1)	82 (62.1)	
22.5–24.9 kg/m^2^	64 (21.3)	54 (22.0)	21 (15.9)	
≥25 kg/m^2^	57 (19.0)	25 (10.2)	15 (11.4)	
Menarche-*n* (%)				0.377
<12 year	14 (4.7)	17 (6.9)	9 (6.8)	
12–13 year	170 (56.7)	150 (61.2)	72 (54.5)	
≥14 year	116 (38.7)	78 (31.8)	51 (38.6)	
Cycle length-*n* (%)				<0.001
≤ 24 days	4 (1.3)	8 (3.3)	2 (1.5)	
25–29 days	124 (41.3)	122 (49.8)	82 (62.1)	
30–34 days	103 (34.3)	90 (36.7)	42 (31.8)	
≥35 days or irregular	69 (23.0)	25 (10.2)	6 (4.5)	
Length of menstruation-*n* (%)				0.038
≤ 4 days	31 (10.3)	34 (13.9)	8 (6.1)	
5–6 days	187 (62.3)	132 (53.9)	74 (56.1)	
≥7 days	82 (27.3)	79 (32.2)	50 (37.9)	
Duration of infertility-n (%)				0.193
<2 years	63 (21.0)	46 (18.8)	38 (28.8)	
2–3 years	139 (46.3)	116 (47.3)	60 (45.5)	
>3 years	98 (32.7)	83 (33.9)	34 (25.8)	
Gravida-*n* (%)				0.031
0	164 (54.7)	143 (58.4)	90 (68.2)	
≥1	136 (45.3)	102 (41.6)	42 (31.8)	
Parity-*n* (%)				0.029
0	272 (90.7)	236 (96.3)	124 (93.9)	
≥1	28 (9.3)	9 (3.7)	8 (6.1)	
Pain^a^ -*n* (%)	39 (13.0)	46 (18.8)	52 (39.4)	<0.001
Palpable nodularity^b^ -*n* (%)	9 (3.0)	24 (9.8)	36 (27.3)	<0.001
Endometrioma diagnosed on TVS-*n* (%)	2 (0.7)	6 (2.4)	73 (55.3)	<0.001
Tubal pathology diagnosed on HSG*-n* (%)	273 (91.0)	216 (88.2)	92 (69.7)	<0.001
Tubal pathology-*n* (%)				<0.001
Bilateral normal	140 (46.7)	179 (73.1)	88 (66.7)	
Unilateral proximal tubal occlusion	31 (10.3)	23 (9.4)	15 (11.4)	
Bilateral proximal tubal occlusion	14 (4.7)	6 (2.4)	2 (1.5)	
Unilateral distal tubal occlusion	53 (17.7)	25 (10.2)	19 (14.4)	
Unilateral distal tubal occlusion, contra-lateral proximal tubal occlusion	19 (6.3)	6 (2.4)	2 (1.5)	
Bilateral distal tubal occlusion	43 (14.3)	6 (2.4)	6 (4.5)	

*BMI, body mass index; SD, Standard Deviation; TVS, transvaginal sonography*.

a *Presence of dysmenorrhea with need for analgesic medication most of the time, and/or intermenstrual pelvic pain, and/or dyspareunia*.

b *Presence of a palpable nodularity in pouch of Douglas on bimanual examination*.

**Figure 2:**
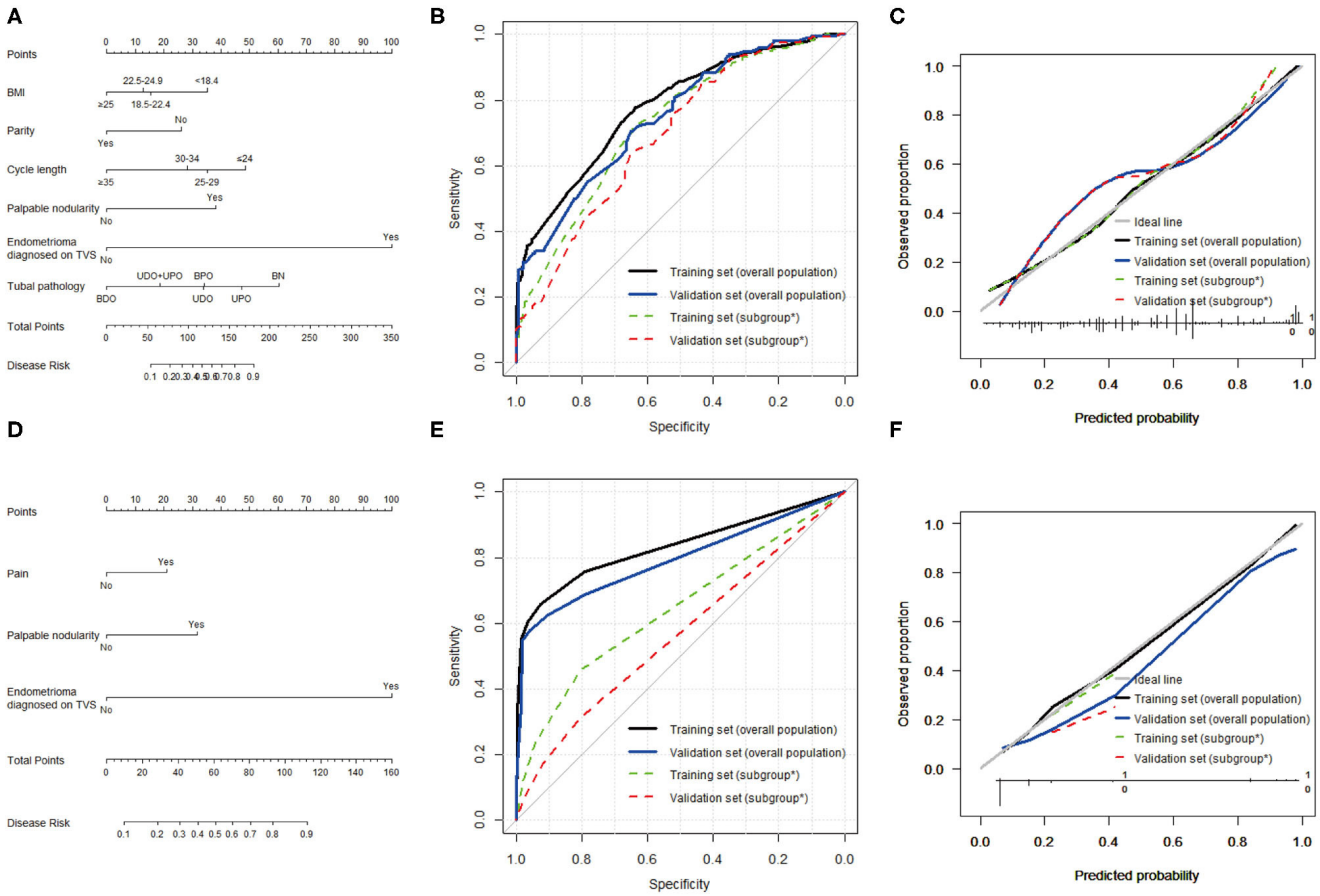
Predictive models for any-stage endometriosis and stage III–IV endometriosis. **(A)** Nomogram, **(B)** ROC curve, and **(C)** calibration plot to predict any-stage endometriosis. This model was based on BMI, menstrual cycle length, parity, palpable nodularity, endometrioma, and tubal pathology. **(D)** Nomogram, **(E)** ROC curve, and **(F)** calibration plot to predict stage III–IV endometriosis. This model was based on pain, palpable nodularity, and endometrioma. *Subgroup of patients without endometrioma diagnosed by TVS.

Six variables were included in the model for any-stage endometriosis after a backward, stepwise selection procedure: BMI, parity, cycle length, palpable nodularity, endometrioma diagnosed on TVS, and tubal pathology. In the model for endometriosis stage III–IV, three variables were selected: pain, palpable nodularity, endometrioma diagnosed on TVS. The nomograms for both models are reported in Figure 2, and the results of each model tested are shown in Table 3.

**Table 3:** Validation of the models for any stage endometriosis and stage III–IV endometriosis.

**Model**		**Any stage endometriosis**				**Stage III–IV endometriosis**			
Variables		BMI, Cycle length, Parity, Palpable nodularity, Endometrioma diagnosed on TVS, Tubal pathology				Pain, Palpable nodularity, Endometrioma diagnosed on TVS			
Patients		Overall population		Subgroup of patients without endometrioma diagnosed by TVS		Overall population		Subgroup of patients without endometrioma diagnosed by TVS	
		Training sample *N* = 677	Validation sample *N* = 339	Training sample *N* = 596	Validation sample *N* = 299	Training sample *N* = 677	Validation sample *N* = 339	Training sample *N* = 596	Validation sample *N* = 299
Discrimination	AUC (95% CI)	0.780 (0.746–0.814)	0.750 (0.699–0.801)	0.726 (0.686–0.766)	0.694 (0.635–0.753)	0.833 (0.789–0.877)	0.793 (0.725–0.860)	0.642 (0.572–0.711)	0.564 (0.472–0.656)
	*P*-value^*^	0.34		0.38		0.33		0.18	
Calibration	*P*-value of unreliability index	1.0	0.094	0.99	0.10	1.0	0.27	1.0	0.27
	E aver	1.2%	6.0%	1.5%	6.4%	0.28%	2.8%	NA	NA
	E max	1.0*10^−7^%	8.3%	0.40%	8.6%	5.4*10^−7^%	10.7%	0.85%	37.2%

* *For AUC comparison. AUC, area under the ROC curve; CI, confidence interval; E, difference in predicted probability and observed frequencies; E aver, average error. E max, maximal error*.

In the training sample, the nomogram for any-stage endometriosis had an AUC of 0.780 [95% confidence interval [CI], 0.746–0.814] in the overall population and 0.726 (95% CI, 0.686–0.766) in the subgroup that was negative for endometrioma diagnosed on TVS. The calibration was good with no significant maximal and average differences between the predicted probabilities and the observed frequencies. In the validation sample, the nomogram for any-stage endometriosis has an AUC of 0.750 (95% CI, 0.699–0.801) in the overall population and 0.694 (95% CI, 0.635–0.753) in the subgroup. The calibration was acceptable with average and maximal errors of 8.3 and 8.6%, respectively in the overall populations, and 6.0 and 6.4%, respectively, in the subgroups. The ROC curve and calibration plot are given in Figure 2.

In the overall population, the AUCs of the nomogram for endometriosis stage III–IV are 0.833 (95% CI, 0.789–0.877) in the training sample and 0.793 (95% CI, 0.725–0.860) in the validation sample. The nomograms were well-calibrated. However, the AUCs and calibration of the nomogram for endometriosis stage III–IV in the subset were unsatisfactory. The ROC curve and calibration plots are given in Figure 2.

The differences in the predictive performances of each model when the variables were changed are shown in Table 4. The AUCs of the full models for any-stage endometriosis were 0.792 (95% CI, 0.759–0.825) in the training sample and 0.760 (95% CI, 0.710–0.810) in the validation sample. The AUCs of the full models were profoundly affected by removing endometrioma or tubal pathology as a variable both in the training and validation samples. The patients' history and symptom, endometrioma, and tubal pathology contributed to the calibration of the nomograms. The AUCs of the model built by endometrioma on TVS for any-stage endometriosis were 0.601 (95% CI, 0.580–0.623) in the training sample and 0.596 (95%CI 0.567–0.625) in the validation sample. The AUCs of the full models for stage III–IV endometriosis were 0.885 (95% CI 0.848–0.922) and 0.799 (95%CI 0.723–0.875) for the training and validation samples, respectively. The AUC and IDI of the full model were profoundly affected by removing endometrioma as a variable. Removing tubal pathology did not significantly affect the performance of the model in the training sample. The AUCs of the model built by endometrioma on TVS only for stage III–IV endometriosis was 0.769 (95% CI, 0.726–0.812) in the training sample and 0.764 (95% CI, 0.702–0.826) in the validation sample, indicating that stage III–IV endometriosis can be reliably predicted using imaging.

**Table 4:** Comparison of receiver operating characteristics curves of the different models predicting any-stage and stage III/IV endometriosis.

	**Training sample**							**Validation sample**						
	**AUC**	**95% CI**	Z Score^*^	*P*-value^*^	**IDI, %**	**95% CI**	***P*-value**	**AUC**	**95% CI**	Z Score^*^	*P*-value^*^	**IDI, %**	**95% CI**	***P*-value**
**Any-stage endometriosis**														
Full model	0.792	0.759–0.825	–	–	–	–	–	0.760	0.710–0.810					
Full model no patients' history and symptom	0.730	0.696–0.764	−4.58	<0.001	−7.18	−9.18/−5.19	<0.001	0.735	0.686–0.783	−1.46	0.14	−4.7	−7.47/−1.94	<0.001
Full model no palpable nodularity	0.783	0.749–0.816	−1.94	0.052	−1.25	−2.04/−0.45	0.002	0.763	0.712–0.813	0.34	0.73	−0.75	−2.1/−0.61	0.28
Full model no endometrioma diagnosed by TVS	0.759	0.723–0.795	−4.33	<0.001	−5.31	−6.88/−3.74	<0.001	0.734	0.681–0.787	−3.14	0.002	−4.37	−6.24/−2.5	<0.001
Full model no tubal pathology	0.753	0.718–0.789	−3.18	0.001	−6.20	−8.13/−4.27	<0.001	0.690	0.635–0.746	−3.67	<0.001	−8.87	−11.91/−5.83	<0.001
Endometrioma diagnosed on TVS only	0.601	0.580–0.623	−11.57	<0.001	−16.4	−19.28/−13.53	<0.001	0.596	0.567–0.625	−6.69	<0.001	−15.58	−19.92/−11.23	<0.001
**Stage III–IV endometriosis**														
Full model	0.885	0.848–0.922	–	–	–	–	–	0.799	0.723–0.875	–	–	–	–	–
Full model no patient history and symptom	0.824	0.777–0.871	−4.08	<0.001	−1.73	−2.81/−0.64	0.002	0.780	0.702–0.857	−0.81	0.42	−2.49	−4.01/−0.97	0.001
Full model no palpable nodularity	0.870	0.830–0.909	−1.76	0.08	−1.03	−1.80/−0.25	0.009	0.798	0.724–0.872	−0.12	0.90	−1.20	−2.47/−0.07	0.064
Full model no endometrioma diagnosed by TVS	0.779	0.734–0.824	−6.11	<0.001	−9.50	−12.45/−6.54	<0.001	0.705	0.631–0.780	−3.77	<0.001	−7.66	−11.71/−3.61	<0.001
Full model no tubal pathology	0.886	0.850–0.921	0.15	0.88	−0.48	−0.86/−0.10	0.014	0.799	0.723–0.875	−0.065	0.95	−0.41	−0.93/−0.10	0.12
Endometrioma diagnosed on TVS only	0.769	0.726–0.812	−6.19	<0.001	−3.99	−5.35/−2.63	<0.001	0.764	0.702–0.826	−1.22	0.22	−4.39	−6.41/−2.36	<0.001

* *For AUC comparison. AUC, area under the ROC curve; CI, confidence interval; IDI, integrated discrimination improvement*.

## Discussion

We developed nomograms to predict any-stage and stage III–IV endometriosis in infertile women and validated the performances of nomograms in all participants and in a subgroup without endometrioma on TVS. Additionally, the effects of variables on the predictive ability of these models were evaluated.

The prevalence of endometriosis in our study population and the characteristics of patients with endometriosis were mostly consistent with previous reports. Endometriosis was present in 56.4% of our patients, which is slightly higher than previously reported (3). This may be due to the fact that the patients undergoing laparoscopy were not randomly selected from an infertile population (5), and that the diagnosis of endometriosis in this study was based on visual evidence alone. As previously reported, endometriosis is inversely associated with BMI (3, 16, 21) and the length of cycles (22). The incidence is higher in primary infertile and nulliparous women (5). However, patients with stage III–IV endometriosis had a longer average menstruation in our study, which is not consistent with previous reports (17). Moreover, we did not detect an increased risk of endometriosis in patients with a later menarche, or a short duration of infertility (17). Pain, despite being defined differently from the previous definition, was found to be an important predictor in our study (23). A study showed that the incidence rate of endometrioma found by TVS was reported to be 3/73 in infertile women without endometriosis and 1/44 in women with stage I-II endometriosis, which is consistent with our results (9). Tubal pathology was originally included as a predictor in our study. Tubal abnormality is one of important reason for infertility, and can be caused by infections, previous surgery, or endometriosis. Approximately 50% of patients in the non-endometriosis group had tubal abnormalities which is much higher than that in the endometriosis group, although endometriosis was also reported as a risk for tuboperitoneal pathology (24). This is probably due to HSG is useful in ruling out tubal blockage but has limited diagnostic value for the peritubal adhesions that are often found in endometriosis (25, 26).

Any-stage and stage III-IV models showed differences with regard to predictive factors and predictive performance in different populations. Six variables were included in the model for any-stage endometriosis, and three variables were included in the model for stage III-IV endometriosis (Table 3). Our model for predicting any-stage endometriosis has good discrimination, with an AUC of 0.780 in the training sample and 0.750 in the validation sample. The model developed to predict stage III–IV endometriosis also has good discrimination, with an AUC of 0.833 in the training sample and 0.793 in the validation sample. These models were well-calibrated, with no significant differences between the predicted and the observed probabilities. To further eliminate the predictive performance of the nomograms, a subgroup of patients without endometrioma diagnosed on TVS, which comprised of approximately 90% of patients, was specifically analyzed. This is because these patients were less likely to be considered for endometriosis before surgery. However, the predictive performance of both models decreased profoundly in the subgroup. The nomogram predicting any-stage endometriosis in the subgroup had an AUC of 0.726 in the training sample and 0.694 in the validation sample, with good calibration. The performance was just fair. The nomogram predicting stage III–IV performance was poor in the subgroup.

To better understand the impact of different variables and different performances of predictive models on different populations, variables were removed from each model one at a time and the resulting models were analyzed. The models built by the variable of endometrioma diagnosed on TVS were analyzed separately (Table 4). When removing patients' history and symptoms, the discriminations of full models for any-stage and stage III–IV endometriosis were not significantly affected in the validation sample. However, we noticed that the calibrations were profoundly affected which indicated its potential contribution to the accuracy of the models. There are numerous predictive models aiming to predict endometriosis based on the patients' history and symptoms. However, their performances were either fair (5, 9), or the model included a history of benign ovarian cysts and surgery/consultation for ovarian cyst as important variables that need to be diagnosed by imaging (27). Comparatively, removing palpable nodularity as a variable did not affect the model significantly. It is likely due to the fact that palpable nodularity on PV is not sensitive for predicting endometriosis, and detecting pouch of Douglas (POD) obliteration and deep infiltrating endometriosis (DIE) of the rectum is not standard on our ultrasound reports. It has been reported that the sensitivity of POD obliteration on PV is 70%, but can be improved to 87% when combined with TVS (13). The POD obliteration and DIE of the rectum were also present in 1/4 and 1/10 of the cases with endometriosis without endometrioma, respectively (28). Thus, detection of POD obliteration and DIE of the rectum with TVS can potentially significantly improve the performance of predictive models on the subgroup without endometrioma. Eliminating endometrioma profoundly worsened the discrimination and calibration in the models which were developed to predict any stage and stage III–IV endometriosis. Endometrioma diagnosed on TVS is reported to have good sensitivity and excellent specificity (13, 29), and stage III–IV endometriosis is reliably predicted using endometrioma on TVS only. Tubal pathology diagnosed on HSG is a variable specific to infertility. The variable strongly affected the discrimination and calibration of the predictive model for any stage endometriosis. However, HSG had little effects on predicting stage III–IV endometriosis. This is likely due to the fact that this divergence was neutralized by including stage I–II endometriosis as controls and because stage III–IV endometriosis typically has more tubal abnormalities.

This study proved the importance of endometrioma on TVS as a variable in predicting endometriosis, and speculated that the detection of POD obliteration and DIE of the rectum with TVS is a promising way to improve the predictive ability of models for its accuracy. Clinical diagnosis of endometriosis is vital as it may reduce the delay in time to diagnosis. However, it is inconsistent, and, currently, there is no common standard diagnostic protocol (6). Patients' history and symptoms, physical examinations, and images are identified in clinics to preoperatively diagnose endometriosis (6), but the role of these variables in predictive models are seldom analyzed. We demonstrated these variables' impacts on models predicting any-stage and stage III–IV models in our study and stressed the importance of imaging in predicting endometriosis. The importance of imaging does not diminish the value of other variables in predicting endometriosis, which could modify models and improve predictive performance. As the POD and DIE of the rectum identified by TVS were not included as a variable in our study, we recommend that this study be used as encouragement to advance the utilization of advanced imaging for better clinical prognosis. It has been proven to be easily proficient in the diagnosis of POD and DIE of the rectum by sonographers who are familiar with the general use of TVS (30).

This study is not without limitations. First, the retrospective nature of this study cannot exclude all potential biases. The variables included in this study were recorded in a consistent manner in all of the patients to reduce bias caused by unrecorded information, which could result in the bias that some important variables might have been missed in our study. Second, infertile patients undergoing laparoscopy is a unique patient population which limits the generalizability of these results. The patients included may have had a propensity to undergo surgery, increasing their pre-test probability of endometriosis. Patients that underwent IVF instead of surgery might distort this result. Third, the POD and DIE of the rectum identified by TVS were not included as variables, which, if included, could have improved the performance of the models. Fourth, in patients without tubal pathology diagnosed by HSG, tubal testing findings at laparoscopy were used instead. Fifth, an external validation of our predictive models is required.

In conclusion, we developed two nomograms that can predict any-stage and stage III–IV endometriosis in infertile women. These nomograms performed well in all participants, but the performance was significantly decreased in a subgroup without endometrioma. Imaging has been proven to be important for predicting endometriosis. Therefore, we recommend that this study be used as encouragement to advance the utilization of advanced imaging to better diagnose and predict endometriosis.

## Data Availability Statement

The datasets presented in this article are not readily available because all the clinic information is generated from the HIS system of our hospital. It is patients' privacy and we should treat them confidentially. Requests to access the datasets should be directed to yuqi2008001@sina.com.

## Ethics Statement

The studies involving human participants were reviewed and approved by Ethics Committee of Peking Union Medical College Hospital. The patients/participants provided their written informed consent to participate in this study.

## Author Contributions

ZG and QY designed the study. ZG, XC, and RT acquired the data. ZG and PF performed the statistical analyses. ZG wrote the manuscript and submitted the manuscript. QY revised the paper. All authors approved the final version.

## Conflict of Interest

The authors declare that the research was conducted in the absence of any commercial or financial relationships that could be construed as a potential conflict of interest.
